# The Berlin index of health and social deprivation: a data based tool to tackle health inequality

**DOI:** 10.1093/eurpub/ckac129.031

**Published:** 2022-10-25

**Authors:** J Zeiher, K Häßler, JD Finger, A Haase, S Hermann

**Affiliations:** Department of Health, Senate Department for Health, Berlin, Germany

## Abstract

**Background:**

Reducing socially induced health inequalities is a key task of urban and regional public health authorities. The Berlin index of health and social deprivation (BIHSD) 2022 aims to show regional differences in health and social situation in sub-areas of the city, to observe developments over time and to identify socially deprived sub-areas of the city.

**Methods:**

The BIHSD 2022 is based on 20 indicators, most of which come from official statistics. Principal component analyses were applied to calculate subindices for the dimensions employment (e.g. unemployment rate), social conditions (e.g. risk-of-poverty rate) and health (e.g. premature mortality). Based on these subindices the final health and social index was derived. The (sub)indices are available on different spatial levels. Relative changes compared to the BIHSD 2013 were calculated to identify regional trends in the transitions in the social structure and health of the city over time.

**Results:**

Besides improvements for most indicators over time in most regions of Berlin, there is still significant evidence for health and social inequality across the city. For example, long-term unemployment rate varies between 0.5% and 40.4% on the lowest spatial level. Following a secular trend, there are substantial improvements in former deprived areas in the inner city while in many peripheral residential areas with an average social structure in the past a downwards trend was observed.

**Conclusions:**

Deprivation indices are helpful tools for research and health reporting in providing evidence for regional inequality. Additionally, they can be used to tailor health promotion strategies and to promote a targeted allocation of financial resources. For example, results of the BIHSD 2022 are being used in epidemiological analyses (e.g. regional inequalities in the risk of SARS-CoV-2 infection) and for (health) policy planning (e.g. needs- and demand-based planning of healthcare).

**Key messages:**

• The Berlin index of health and social deprivation 2022 show regional differences in health and social situation in sub-areas of the city and documents developments over time.

• The index is being used in epidemiological analyses, to tailor health promotion strategies, and to promote a targeted allocation of financial resources.

